# Interaction of Host Nucleolin with Influenza A Virus Nucleoprotein in the Early Phase of Infection Limits the Late Viral Gene Expression

**DOI:** 10.1371/journal.pone.0164146

**Published:** 2016-10-06

**Authors:** Deepshikha Kumar, Shobha Broor, Maitreyi S. Rajala

**Affiliations:** 1 School of Biotechnology, Jawaharlal Nehru University, New Delhi, India; 2 Department of Microbiology, Faculty of Medicine and Health Science, Shree Guru Gobind Singh Tricentenary University, Gurgaon, Haryana, India; University of Alabama at Birmingham, UNITED STATES

## Abstract

Influenza A virus nucleoprotein, is a multifunctional RNA-binding protein, encoded by segment-5 of the negative sense RNA genome. It serves as a key connector between the virus and the host during virus replication. It continuously shuttles between the cytoplasm and the nucleus interacting with various host cellular factors. In the current study, host proteins interacting with nucleoprotein of Influenza A virus of H1N1 2009 pandemic strain were identified by co-immunoprecipitation studies followed by MALDI-TOF/MS analysis. Here we report the host nucleolin, a major RNA-binding protein of the nucleolus as a novel interacting partner to influenza A virus nucleoprotein. We thus, explored the implications of this interaction in virus life cycle and our studies have shown that these two proteins interact early during infection in the cytoplasm of infected cells. Depletion of nucleolin in A549 cells by siRNA targeting endogenous nucleolin followed by influenza A virus infection, disrupted its interaction with viral nucleoprotein, resulting in increased expression of gene transcripts encoding late viral proteins; matrix (M1) and hemagglutinin (HA) in infected cells. On the contrary, over expression of nucleolin in cells transiently transfected with pEGFP-NCL construct followed by virus infection significantly reduced the late viral gene transcripts, and consequently the viral titer. Altered expression of late viral genes and titers following manipulation of host cellular nucleolin, proposes the functional importance of its interaction with nucleoprotein during influenza A virus infection.

## Introduction

Influenza A virus is a public health threat worldwide and contributes to a high-level of mortality during pandemics. Its segmented genetic composition allows re-assortment of gene segments between the strains of different host origins resulting in the emergence of a novel strain and an unpredictable pandemic. Eight negative sense single stranded RNA gene segments of influenza A virus encode for 10 proteins. However, 7 more novel proteins; PB1-F2 [1], PB1-N40 [2], PA-N155, PA-N182 [3], PA-X [4], M42 [5] and NS3 [6] were discovered 40years after the genome mapping of influenza A virus was done. Nucleoprotein (NP), a 56kDa protein, encoded by segment-5, is a multifunctional RNA binding protein. The primary function of this protein is to encapsidate the viral genome and form homo-oligomers to maintain ribonucleoprotein complex (RNP) structure [7]. It acts as a cofactor to coat the newly synthesized viral complementary RNA [8]. Also, NP determines whether complementary RNA, synthesized from genomic RNA, is to be used for protein translation or to serve as a template for synthesis of genomic strand during the replication [9]. Besides its active role in the replication of virus, NP contributes to host adaptation when avian strains change their host to mammalian species. During adaptation, mutations in NP together with subunits of RNA polymerase contribute to increased polymerase activity of avian strains [10]. All the above reports together signify the indispensable role of NP in influenza A virus life cycle.

Owing to their limited genetic coding capacity, viruses depend on the host machinery for their survival. The virus and the host protein interactions in infected cell during virus replication determine the outcome of the disease. Interaction of host proteins with influenza A virus proteins have been reported earlier in the literature using various approaches such as genome-wide RNA interference screening [11], yeast-two-hybrid screening [12], comprehensive analysis of influenza virus polymerase cellular interactome [13] and proteome based approaches [14]. As influenza virus replicates inside the nucleus, NP continuously shuttles between the nucleus and the cytoplasm interacting with various host factors to regulate multiple functions during the replication [15]. Viral RNP complex is imported into the nucleus through its interaction with importin α [16], and viral RNA synthesis is regulated by interaction between NP and cellular factors such as BAT1/UAP56, Tat-SF1, MCM complexes [17, 18, 19]. So is export of vRNA into the cytoplasm mediated by NP interaction with CRM1 [20]. Furthermore, NP association with cytoskeletal proteins such as alpha actinin-4 demonstrated to be essential for its translocation into the nucleus [21]. Nonetheless, the host interacting partners to NP of 2009 pandemic H1N1 strain and the interacting partners that are common to NP of circulating and emerging strains of influenza A virus are largely unknown. Since, the gene encoding viral NP is conserved among the strains isolated from different hosts with less than 11% of the amino acid difference [22], NP of different influenza A viral strains are expected to have common host interacting partners.

In the current study, we sought to identify the novel host interacting partners of influenza A virus NP of 2009 H1N1 pandemic strain and validate the identified interactions in cells infected with seasonal strains like H1N1 and H3N2. Total NP of each subcellular protein fraction recovered from cells expressing the recombinant viral NP of 2009 pandemic H1N1 strain was co-immunoprecipitated with anti-Myc antibody. Proteins co-precipitated with the recombinant viral NP were identified by mass spectrometric analysis using matrix-assisted laser desorption ionization-time of flight (MALDI-TOF/MS analysis). Host cellular nucleolin, a multifunctional and an abundant protein of nucleolus was identified to be one of the novel host interacting partners to recombinant viral NP in the cytosolic fraction. Interaction of host nucleolin with viral NP was confirmed in A549 cells infected with pandemic or seasonal strains. Various experimental approaches were used to evaluate the interplay between the host nucleolin and the viral NP in cells infected with pandemic H1N1 strain.

Our results demonstrate that the host nucleolin interaction with viral NP reduces the viral titer by limiting the transcription of late viral genes. Nonetheless, the mechanism involved in the regulation of late viral gene transcription through nucleolin and NP interaction is yet to be characterized.

 

## Material and Methods

### Cell lines and viruses

A549 (Human lung adenocarcinoma epithelial cells) and MDCK (Madin-Darby Canine Kidney) cell lines were obtained from National Centre for Cell Science (Cell line repository), Pune, India. A549 cell line was used for transfection and infection experiments while MDCK for propagation of virus [23, 24]. A549 and MDCK cells were maintained in Ham’s Nutrient Mixture F12 and DMEM supplemented with 10% FBS and Penicillin-Streptomycin solution respectively. Cells were maintained at 37°C in a 5% CO_2_ atmosphere. Culture reagents were obtained from Hi-Media Laboratories. Influenza A virus of 2009 H1N1 pandemic and seasonal strains (H3N2, H1N1) isolated from patients at AIIMS, New Delhi were used in the study.

### Virus infection and propagation

MDCK cells were washed twice with PBS (pH7.2) and incubated for 1hr with 100μl of inoculums from patient samples at 37°C in 5% CO_2_ atmosphere for adsorption. After 1hr, inoculum was decanted and infected cells were supplemented with DMEM containing 0.2% BSA, 20mM HEPES (pH7.2) and 1μg/ml TPCK (Sigma-Aldrich). Incubation was continued until the manifestation of virus induced CPE. Infected cells were harvested and subjected to repeated freeze-thaw cycles. Viral cultures were stored at -80°C until further use and the titer was determined by TCID_50_ assay. For viral and host protein interaction studies, virus infections were done in A549 cells.

### Antibodies

Anti-nucleolin C23 (H-250) polyclonal, anti-influenza A viral M1 (FluAc) monoclonal and β-actin (C-4) monoclonal antibodies were obtained from Santa Cruz Biotechnology, USA. Anti-influenza A virus nucleoprotein monoclonal (H16-L10-4R5) antibody was obtained from Merck Millipore. Anti-Myc (9E10), anti-polyhistidine (His-1) mouse monoclonal antibodies, anti-mouse IgG (whole molecule) FITC, anti-rabbit IgG F (ab2) fragment TRITC, anti-mouse (whole molecule) peroxidase (A4416), and anti-rabbit (whole molecule) peroxidase (A6154) antibodies were obtained from Sigma-Aldrich, USA. For western blotting of viral NP, antiserum raised in Swiss Albino mice against purified recombinant viral NP (pET29a+NP) was used.

### Recombinant constructs

Total RNA was isolated from cells infected with influenza A virus of 2009 pandemic H1N1 strain using Trizol reagent following manufacturer’s instructions. A fraction of total RNA was reverse transcribed with random hexamer (pdn6), reverse transcriptase enzyme; MMLV. Complementary DNA was amplified using NP gene specific primers (FP: 5’ATATGAATTCACCATGGCGTCCCAAGGCACC 3’, RP: 5’ATATCTCGAGTTACAGATCTTCAGAAATA AGTTTTTTGTTCATTGTCGTACTCCTCTGCATTG 3’). Amplified product was purified and cloned into eukaryotic expression vector pcDNA3.1(+) driven by CMV promoter between *Eco* RI and *Xho* I restriction sites with Myc epitope tag at the C-terminal coding region of the target gene.

### Transfection and subcellular fractionation

A549 cells were transiently transfected with the recombinant viral NP and mock transfected with the empty vector, pcDNA 3.1(+) using Lipofectamine LTX reagent (Invitrogen^TM^) following standard protocol. The recombinant protein expression was confirmed by IFA staining and western blotting using anti-viral NP antibody. Subcellular fractionation of transfected cells was done using a protocol developed by Guimaraes de Araujo G et al., [25]. In brief, transfected cells were washed and scrapped into ice-cold 1×PBS (pH7.4) and centrifuged at 700× g for 5 min at 4°C. Pellet was resuspended in HB buffer (250mM sucrose, 3mM imidazole (pH 7.4), 1mM EDTA, 1× protease inhibitors) and centrifuged at 1100×g for 10 min at 4°C. The resultant pellet was resuspended in HB buffer, homogenized and centrifuged at 1600×g for 10 min at 4°C. Pellet had the nuclear fraction while supernatant had the membrane and cytosolic fractions. From the supernatant, membrane and cytosolic fractions were separated by ultracentrifugation for 1hr at 100000×g at 4°C. Supernatant containing cytosolic fraction was collected in a fresh tube and the pellet containing membrane fraction was resuspended in HB buffer containing 1% Triton X-100. Pellet with the nuclear fraction from the earlier step was resuspended in TSE buffer (100mM Tris, 300mM sucrose, 1mM EDTA, 0.1% IGEPAL-CA 630 v/v, (pH 7.5)) and centrifuged at 4000×g for 5 min at 4°C. The resultant pellet was washed with TSE buffer till the supernatant was clear and resuspended in TSE buffer to obtain the final nuclear protein fraction. Distribution of proteins in each fraction was confirmed by western blotting using antibodies against markers specific to each fraction. The total protein concentration in each fraction was determined by Bradford assay.

### MALDI-TOF/Mass spectrometry analysis

Two hundred μg of total protein from each subcellular fraction was pre cleared by incubation with 30μl of protein A agarose beads for 2hrs at 4°C followed by centrifugation at 3000rpm. Cleared supernatants were co-immunoprecipitated with anti-Myc antibody as the recombinant viral NP construct has a Myc epitope tag at its C-terminus. Lysates with anti-Myc antibody were incubated overnight at 4°C. Then, 50μl of protein A agarose beads was added and incubated for an additional 2hrs at 4°C. Immune complexes were washed thrice with chilled NP-40 buffer (20mM Tris-Cl pH 7.4, 100mM NaCl, 10% Glycerol, 1% NP-40, 1mM EDTA, protease inhibitors), solubilized in 2×SDS sample buffer and boiled for 5min. Insoluble material was removed by brief centrifugation and subjected to SDS-PAGE to resolve the host cellular proteins co-precipitated with the recombinant viral NP. Gels were silver stained and protein bands visualized in immune complexes prepared from cells expressing the recombinant viral NP but not the vector containing cells were analyzed by MALDI-TOF/MS analysis. Corresponding bands were excised from silver stained gels and subjected to tryptic digestion. Digested peptides were loaded on to the proteome analyzer SCIEX TOF/TOF 5800 (Applied Bio systems). Peptides were analyzed by mass spectrometry using MALDI-TOF. Identified peptides were searched against MASCOT database using the Protein Pilot software (AB SCIEX) connected to its server.

### Co-immunoprecipitation

Host nucleolin that was identified as one of the putative interacting partners to viral NP by MALDI-TOF/MS analysis was further evaluated by co-immunoprecipitation and co-localization studies. A549 cells were transiently transfected with the recombinant gene encoding viral NP and mock transfected with the empty vector as described above. At 24hrs post transfection, cells were harvested in RIPA buffer containing 10mM Tris-Cl (pH 7.4), 150mM NaCl, 5mM EDTA (pH 8.0), 1% triton-X100, 1% sodium deoxycholate, 0.1% SDS, 1mM PMSF, 1mM sodium orthovanadate, protease inhibitors. Five hundred *μ*g of total protein recovered from transfected cells was co-immunoprecipitated with anti-Myc antibody or anti-nucleolin antibody followed by immunoblotting with anti-nucleolin or anti-Myc antibody. Similarly, A549 cells were infected with influenza A virus 2009 pandemic H1N1 strain at an MOI of 5 and mock infected with serum free medium. At desired time points post infection, cells were harvested and the total protein was recovered. Five hundred μg of total protein from each experimental sample was co-immunoprecipitated with anti-nucleolin antibody and immune complexes were resolved on SDS-PAGE followed by immunoblotting with anti-viral NP antiserum.

### Confocal microscopy

A549 cells grown in 24 well plates containing 12mm coverslips were either transfected with the recombinant viral NP or directly infected with virus at an MOI of 5. At 24hrs post transfection, cells on coverslips were fixed in chilled acetone for 20min. Untransfected but infected cells on coverslips were fixed at every 2hrs and were analyzed up to 12hrs post infection. Coverslips were washed in 1× PBS and subjected to double IFA staining. In brief, cells were incubated with primary antibodies; mouse monoclonal anti-viral NP (1:100) and rabbit polyclonal anti-nucleolin (C23) antibodies (1:100) for 2hrs at RT followed by three washes in 1×PBS. Coverslips were further overlaid with secondary antibodies; FITC conjugated anti-mouse antibody, and TRITC conjugated anti-rabbit antibody, for 1hr at RT followed by three washes. Nuclei were stained using 2.5μg/ml of Hoechst stain (Sigma-Aldrich) for 10min. Coverslips were mounted on glass slides and were visualized on Confocal Laser Scanning Microscope (Olympus FluoviewTM—FV1000) equipped with HeNe laser (488nm), HeNe laser (543nm) and pulse diode laser (408nm). Images were acquired with PLAPON 60X O NA: 1.42 oil immersion objective using FV10SW1.7 software.

### *In vitro* binding assay

To further validate the interaction between nucleolin and NP, *in vitro* binding assay was carried out using E. *coli* lysates expressing recombinant viral NP. In brief, BL-21pLysS cells were transformed with the recombinant viral NP (Gene encoding NP of H1N1 pandemic strain was cloned into pET29a+ vector). Culture was induced with 1.0mM IPTG (Sigma-Aldrich) overnight at 32°C. Next day, bacterial cell lysates were prepared and protein concentration was determined. Hundred and fifty μg of cell lysate was incubated with 100μl of Ni-NTA beads for 6hrs at 4°C. His tagged recombinant viral NP bound to Ni-NTA beads was washed with 1×PBS containing 2mM DTT, 1mM PMSF and 20mM imidazole (pH 8.0). Beads with bound NP were further incubated overnight with 1mg of A549 cell lysate. An unrelated purified bacterial protein of 28kDa was used as a non specific control. Following day, Ni-NTA beads were washed with 1×PBS and then the bound protein complex was eluted; subjected to SDS-PAGE and immunoblotting with anti-nucleolin, anti-polyhistidine and anti-viral NP antiserum. *In vitro* binding assay was optimized using varying concentrations of bacterial lysates containing recombinant viral protein and different amounts of Ni-NTA beads.

### siRNA transfection

A549 cells were transiently transfected with siRNA targeting nucleolin (siRNA-NCL) and non targeting siRNA (siRNA-NT) (Dharmacon, Thermo Scientific) using Dharmafect -1 reagent following manufacturer’s instructions. Depletion of endogenous nucleolin in A549 cells was confirmed by western blot analysis using anti-nucleolin antibody. For co-localization studies, cells grown on coverslips were transfected with siRNA-NCL or siRNA-NT. At 24hrs post transfection, cells were infected with virus at an MOI of 5 and were analyzed at 5, 6 and 7hrs post infection. Cells fixed at desired time points were subjected to double IFA staining as described above and analyzed on confocal microscope. Virus infections in the subsequent experiments were done at 24hrs post transfection with the desired constructs.

### Quantitative real time PCR

A549 cells were transiently transfected with siRNA-NCL or siRNA-NT followed by virus infections. Cells were harvested at 5, 6, 7, 8 and 24hrs post infection; total RNA was isolated using Trizol reagent. One μg of total RNA was reverse transcribed, and complementary DNA was subjected to quantitative real time PCR using SYBR ^®^ green chemistry to measure the gene transcripts encoding late viral proteins; Matrix (M1) and Hemagglutinin (HA) using specific primers; M1-FP: 5’ACCGAGGTCGAAACGACGT 3’, M1-RP: 5’CCAGTCTCTGCGCGATCT C 3’; HA-FP: 5’GGAAAGAAATGCTGGATCTGGTAT 3’, HA-RP: 5’AATGGGAGGCTGGT GTTTATAGC 3’. GAPDH (FP: 5’TGCACCACCAACTGCTTAGC 3’; RP: 5’GGCATGGACT GTGGTCATGAG 3’) was used as an internal control to normalize PCR for the amount of RNA added in the reverse transcription reaction. Comparison of late viral gene expression between cells transfected with siRNA-NCL and siRNA-NT at different time points was done by calculating the ‘n’ fold difference in mRNA abundance using relative quantification, 2^-ΔΔCT^ method [26]. Where ΔC_T =_ C_T_ of target gene−C_T_ of control gene. ΔΔC_T_ = ΔC_T of_ sample 1− ΔC_T_ of sample 2. M1 or HA gene expression in cells transfected with siRNA-NT was taken as a control and the level of expression was considered one for comparison. Similar experiment was carried out to measure M1, HA and NP (NP-FP: 5’AAGCAGATACTGGGCCATAAGG 3’, NP-RP: 5’GAGAATGTAGGCTGCACACTGATC 3’**)** gene transcripts in cells with overexpressed nucleolin. For which, cells were transfected with pEGFP-NCL construct (GFP-Nucleolin was a kind gift from Michael Kastan (Addgene plasmid #28176) [27] or the empty vector pEGFP-C1 followed by virus infection. Expression of M1and HA including NP gene transcripts was measured at 24hrs post infection. Gene expression in untransfected cells infected with virus was used as a control and the expression level was considered one for comparison.

### Viral titers

Virus titer was measured in cells with depleted or overexpressed nucleolin. A549 cells were transfected with siRNA-NCL or siRNA-NT or pEGFP-NCL or pEGFP-C1 vector followed by infection. Untransfected but virus infected cells were used as controls. At 48hrs following infection with visible CPE, cells were harvested; subjected to repeated freeze thaw cycle to collect cell free and cell associated viral progeny. Further, A549 cells were infected with lysates collected from each group of infected cells. Tenfold serially diluted virus collected from each group was added to cultured cells and each dilution was added in 8 wells of a 96 well plate. At 3days post infection, CPE was scored for the presence of virus replication and the titer was determined by TCID_50_ end point dilution assay. TCID_50_ was calculated by Reed and Müench method as described previously [28].

### MTT assay

The percentage of cell viability was measured in A549 cells infected with progeny virus formed in nucleolin depleted or overexpressed cells by MTT assay. In brief, at 3days post infection cells in each well were incubated with 20μl of MTT (3-(4,5-dimethylthiazol-2-yl)-2,5-diphenyl-2H-tetrazolium bromide) reagent prepared at a concentration of 5mg/ml in PBS for 4hrs. Then, MTT reagent was removed and the resulting formazan crystals were solubilized in 200μl of DMSO. The quantity of formazan which is directly proportional to the number of cells viable was measured by recording absorbance at wavelength of 560nm. The percentage of viability was calculated taking mock infected cells as negative control.

### Statistical analysis

Analysis of the data was carried out using Statistical package for social sciences (SPSS) for Windows version 17.0, Released 2008 (SPSS Inc., Chicago, IL). Every experiment including MTT assay was done in biological triplicates. Data are represented as means and standard deviations. In real time PCR, means of triplicate ΔC_T_ values obtained for each group was compared with its corresponding control by student’s t test. In MTT assay, means of percentage of cell viability between any two groups was compared by student’s t test. For viral titers, due to large variation among groups (log dilutions were used), Mann-Whitney U, a non parametric test was used to compare the means of triplicate TCID_50_ values of each group. A p value <0.05 was considered significant.

## Results

### Identification of host cellular proteins interacting with influenza A virus nucleoprotein

To identify the potential host interacting partners to viral NP, a preliminary experiment was done using recombinant clone of influenza A virus gene encoding NP of 2009 H1N1 pandemic strain. Subcellular fractions prepared from A549 cells transiently expressing recombinant viral NP were co- immunoprecipitated with anti-Myc antibody. Immune complexes were resolved on SDS-PAGE and silver stained. Interacting proteins co-precipitated with the recombinant viral NP were identified by MALDI-TOF/MS analysis. Peptide mass fingerprints obtained were applied to a query data base search engine Mascot. Hits obtained for each protein band with significant Mascot score were further matched with the molecular weight of the corresponding protein band on silver stained gels. The best matches were found for β-actin, nucleolin and POTE ankyrin domain family member E (POTEE). All three proteins were identified in immune complexes prepared from cells expressing recombinant NP. Nucleolin and POTEE were identified to be novel interacting partners to recombinant viral NP in cytosolic fraction with a significant protein score. Interaction between host cellular nucleolin, a major RNA binding protein with influenza A virus NP, which is also an RNA binding protein was validated using different experimental approaches. The impact of nucleolin and NP interaction in infected cells on virus replication was further investigated. F-actin had been reported to be an interacting partner to influenza A virus NP by *in vitro* studies [29], but the significance of interaction between β-actin which was identified to be one of the interacting partners to viral NP is yet to be explored, so is its interaction with POTEE protein.

### Host cellular nucleolin as a novel interacting partner to influenza A virus nucleoprotein

**Fig 1:**
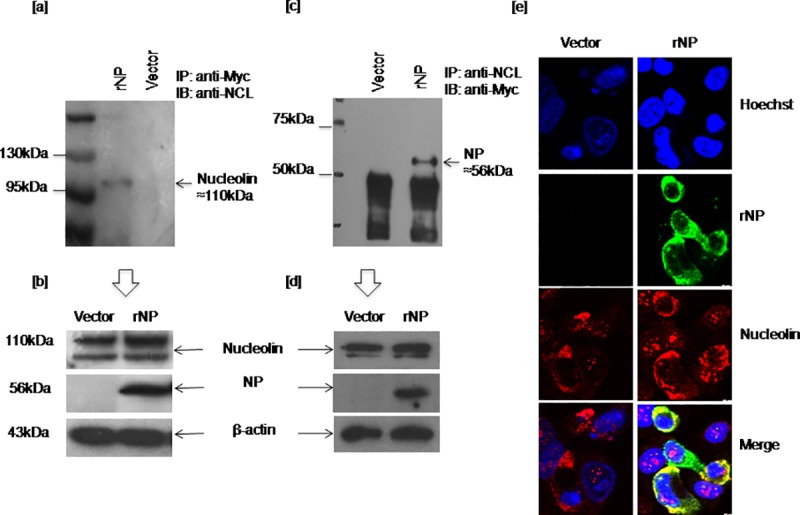
Interaction of recombinant influenza A virus nucleoprotein with host cellular nucleolin. A549 cells were transiently transfected with the recombinant viral NP and mock transfected with the empty vector for co-immunoprecipitation and co-localization studies. Cell lysates prepared at 24hrs post transfection were co-immunoprecipitated with anti-Myc antibody and immune complexes were subjected to immunoblotting using anti-nucleolin antibody. Another transfection experiment was carried out and recovered cell lysates were immunoprecipitated with anti-nucleolin antibody followed by immunoblotting with anti-Myc antibody in a reverse order. Further, cells grown on coverslips were transfected with the recombinant viral NP or the empty vector. Cells were fixed at 24hrs post transfection and subjected to double IFA staining with anti-viral NP (mouse) and anti-nucleolin (rabbit) antibodies. Secondary antibodies used were anti-mouse antibody tagged with fluorophore FITC (green) and anti-rabbit antibody tagged with TRITC (red). [a] Co-precipitation of 110kDa nucleolin protein with recombinant viral NP in A549 cell lysate [b] Expression of viral NP, endogenous nucleolin in transfected cells [c] Co-precipitation (reverse IP) of recombinant viral NP of ~56kDa with nucleolin [d] Expression of viral NP and endogenous nucleolin in the corresponding cell lysates [e] Co-localization of host nucleolin and viral NP (PCC: 0.56 and MOC: 0.82). Nucleus is stained with Hoechst.

To evaluate the nucleolin interaction with viral NP, A549 cells were transiently transfected with the recombinant viral NP or the empty vector. Cell lysates prepared at 24hrs post transfection were co-immunoprecipitated with anti-Myc antibody and immunoblotted with anti-nucleolin antibody. Nucleolin, an110kDa protein was observed to be co-precipitated with viral NP in immune complexes prepared from the recombinant viral NP expressing cells, while no such band was seen in cells transfected with the empty vector, pcDNA 3.1(+) (Fig 1A). Transfection experiment was repeated precisely as above and cell lysates recovered were co-immunoprecipated with anti-nucleolin antibody followed by immunoblotting with anti-Myc antibody in reverse order. Viral NP of ~56kDa was found to be co-precipitated with host nucleolin (Fig 1C). In both the experiments, endogenous nucleolin expression in cells transfected with the recombinant viral NP and the vector was comparable (Fig 1B and 1D). Two experiments independent of each other confirmed the host nucleolin interaction with the recombinant viral NP.

To demonstrate the co-localization of nucleolin and NP, cells grown on coverslips were transfected with the recombinant viral NP and fixed at 24hrs post transfection. Cells were subjected to double IFA staining using primary antibodies to nucleolin, viral NP and secondary antibodies with two different fluorophores. Co-localization of two proteins in cells was visualized by confocal microscope. IFA staining had demonstrated co-localization of nucleolin and recombinant viral NP in the cytoplasm of the cell (Fig 1E). Degree of co-localization between fluorophores was quantified. Correlation measurement was made using Pearson correlation coefficient (PCC) and Mander’s overlap coefficient (MOC) using an image analysis software connected to the confocal microscope. The given confocal image (Fig 1E) had PCC of 0.56 and MOC of 0.82. Both the values indicate the positive correlation. Further, PCC and MOC were measured for few individual cells in the same sample. Co-localization of nucleolin and NP in two individual cells of the same field with PCC values of 0.68, 0.83 and MOC values of 0.83 and 0.89 respectively was shown in the supplementary data (S1 Fig).

### Confirmation of nucleolin and nucleoprotein interaction in cells infected with influenza A virus of 2009 pandemic H1N1 strain

**Fig 2:**
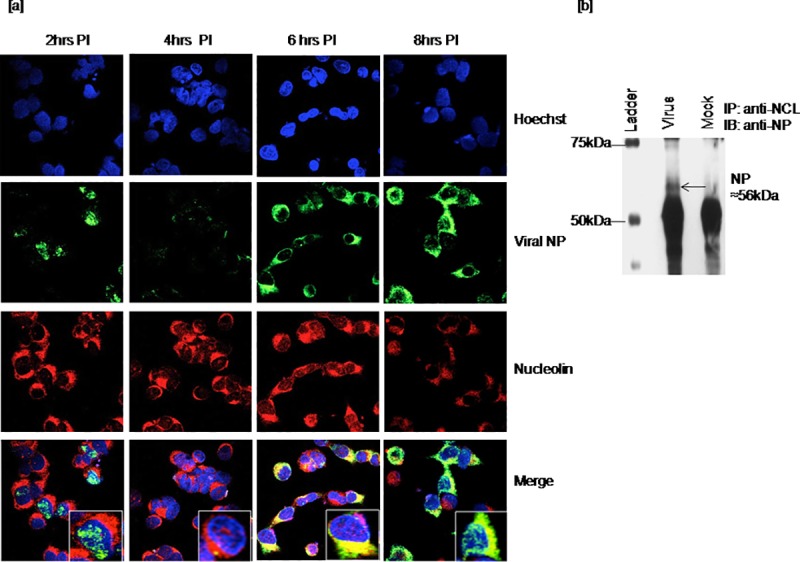
Interaction of viral nucleoprotein with nucleolin in cells infected with influenza A virus of H1N1 2009 pandemic strain. A549 cells grown on coverslips were infected with Influenza A virus and mock infected with serum free medium. Infected cells fixed at every 2hrs up to 12hrs post infection were subjected to double IFA staining with anti-viral NP and anti-nucleolin antibodies as described in the Fig 1. Stained cells were analyzed on confocal microscope. Then, again cell lysate recovered from infected cell at 6hrs post infection was co-immunoprecipitated with anti-nucleolin antibody and immune complexes were subjected to immunoblotting using anti-viral NP antiserum. [a] Co-localization of viral NP and host nucleolin at 6 and 8hrs post infection [b] Co-precipitation of viral NP (56kDa) with host nucleolin. To substantiate the above findings, *in vitro* binding assay was carried out using the recombinant viral NP generated in pET29a+ vector (pET29a+NP). Ni-NTA beads were incubated with BL-21 cells carrying the recombinant viral NP and allowed to immobilize on to Ni-NTA beads. Titration assay was done to determine the quantity of beads and the concentration of bacterial lysate required for optimal *in vitro* binding of nucleolin with the recombinant viral NP. Use of 100μg of protein lysate with different measures of Ni-NTA beads ranging from 12.5 to 100μl had shown visible binding with the use of 100μl beads. Wherein, 100μl of beads incubated with different concentrations of bacterial lysates ranging from 50 to 250μg had demonstrated dose dependent binding (S3 Fig).

To validate the interaction of host nucleolin and viral NP, co-localization and co-precipitation studies were carried in cells directly infected with virus. Influenza A virus usually takes about 6hrs to complete one round of its replication in MDCK cells. However, it does depend on the number of virus particles used for infection [30]. It may also depend on other factors such as virus strain and host cells used for infections. To track the time two proteins associate during infection; A549 cells infected with influenza A virus were analyzed at every 2hrs up to 12hrs post infection. Cells fixed at every 2hrs were subjected to double IFA staining and the association of two proteins was visualized on confocal microscope. Strongest co-localization of viral NP and host nucleolin in the cytoplasm was evident by the merge of green and red, visible in yellow at 6hrs. Degree of co-localization as determined by PCC, 0.59 and MOC, 0.78 revealed positive correlation. Two proteins appeared to be co-localized at 8hrs post infection in the cytoplasm, but PCC of 0.44 and MOC of 0.49 found be relatively lower than the values obtained for 6hrs (Fig 2A). Two proteins were observed to be disassociated from 10 to 12hrs post infection, the last time point in the assay (data not shown). Visualization of co-localized nucleolin with viral NP at 6hrs post infection suggests that the interaction is an early event in virus life cycle. This assay confirmed our preliminary results obtained by MALDI-TOF/MS analysis where nucleolin was found to be co-precipitated with the recombinant viral NP in cytosolic fraction. To further confirm the results, cell lysates prepared from infected cells at 6hrs post infection were subjected to co-immunoprecipitation with anti-nucleolin antibody and immunoblotted with anti-viral NP antiserum. Detection of 56kDa protein band in immune complexes prepared from virus infected cells (Fig 2B) support the co-localization findings.

As the nucleolin-NP interaction observed in the cytoplasm, endogenous nucleolin level in cytosolic and nuclear fraction of infected cells was checked at 4 to 8hrs post infection. For which, equal amount of cytosolic and nuclear protein fractions recovered from virus and mock infected cells was subjected to SDS-PAGE followed by western blotting with anti-nucleolin antibody. The amount of nucleolin protein was found to be relatively high in cytosolic fraction of virus infected cells as compared to mock infected (S2 Fig). Whereas, the nuclear fractions from mock and virus infected cells had shown no obvious difference in the level of nucleolin. The purity of fractions was confirmed by western blotting using antibodies against cytoplasmic and nuclear specific markers; β-actin and c-Jun respectively.

**Fig 3:**
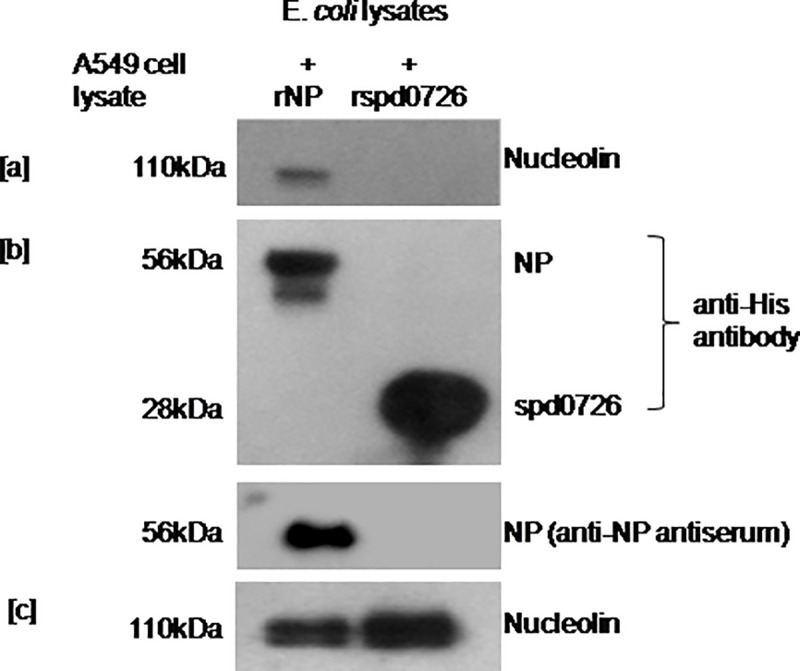
*In vitro* binding assay showing the nucleolin interaction with the recombinant viral nucleoprotein. BL-21 cells were transformed with the recombinant viral NP (pET29a+NP) or unrelated control protein. BL-21 cell lysates were prepared and incubated with Ni-NTA resin for 6hrs. Recombinant viral NP or unrelated protein bound to Ni-NTA beads were washed and further incubated overnight with A549 cells lysates. Next day, beads were washed; bound protein complexes were eluted and subjected to SDS PAGE followed by immunoblotting with anti-nucleolin, anti-His antibody and anti-viral NP antiserum. Cell lysates recovered after centrifugation following incubation with the recombinant viral NP and control protein bound Ni-NTA beads were analyzed for endogenous nucleolin expression [a] Binding of nucleolin, an110kDa protein with the recombinant viral NP, not with the control protein [b] Expression of recombinant viral NP (~56kDa) and unrelated bacterial protein (~28kDa) in BL-21 cell lysates used for the assay with anti-His and anti-viral NP antibodies [c] Endogenous nucleolin expression in A549 cell lysates.

Mixing of beads with bound NP and A549 cell lysates had demonstrated host nucleolin, an 110kDa as an interacting partner to virus NP (Fig 3A). No such interaction was noted with unrelated bacterial protein which was used as a non specific control. Expression of the recombinant viral NP (56kDa) and a non specific recombinant protein (28kDa) in BL-21 cell lysates was confirmed using anti-His antibody and anti-viral NP antiserum (Fig 3B). Endogenous nucleolin level in A549 cell lysates (supernatants) removed after centrifugation following overnight incubation with Ni-NTA beads with bound recombinant viral NP or control protein was comparable (Fig 3C).

### Disruption of nucleolin and nucleoprotein interaction in A549 cells with siRNA targeting nucleolin

**Fig 4:**
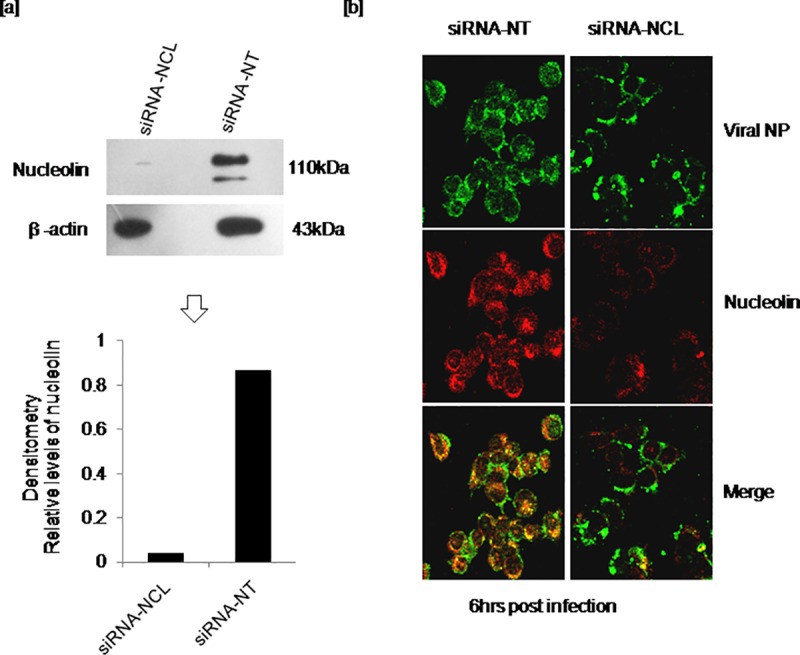
Confirmation of viral nucleoprotein interaction with host nucleolin using siRNA strategy. A549 cells were transfected with siRNA-NCL or siRNA-NT followed by infections. Infected cells were fixed at indicated time points and subjected to double IFA staining using anti-nucleolin, anti-viral NP antibodies and secondary antibodies with two fluorophores. [a] Reduced expression of endogenous nucleolin in cells transfected with siRNA-NCL in comparison with the control construct siRNA-NT [b] Disruption of nucleolin and NP interaction in cells transfected with siRNA-NCL and co-localization of two proteins in cells with siRNA-NT at 6hrs post infection.

To determine the importance of host nucleolin and viral NP interaction during replication, endogenous nucleolin of A549 cells was transiently down-regulated using siRNA-NCL. Cells transfected with siRNA-NT were used as control. Depletion of endogenous nucleolin was marked at 24hrs post transfection and the reduction was about >70% as determined by densitometry analysis (Fig 4A). However, the reduced level of nucleolin did not affect the cell viability. To further investigate whether depletion of endogenous nucleolin restrains its interaction with viral NP, A549 cells with depleted endogenous nucleolin following 24hrs transfection with siRNA-NCL or control construct siRNA-NT were infected with virus. As our previous results demonstrated the interaction of nucleolin and NP at 6hrs post infection, infected cells were subjected to IFA staining at 5 to 7hrs post infection. Co-localization studies had shown no visible interaction between nucleolin and NP in cells with depleted endogenous nucleolin up to 7hrs. However, notable association of these two proteins was observed in cells transfected with siRNA-NT at 6hrs post infection in the cytoplasm (Fig 4B). In accordance with the previous results, the difference observed in cells transfected with siRNA-NCL or siRNA-NT indicates that the host nucleolin does interact with viral NP during infection.

### Host cellular nucleolin and viral nucleoprotein interaction regulates the late viral gene expression

**Fig 5:**
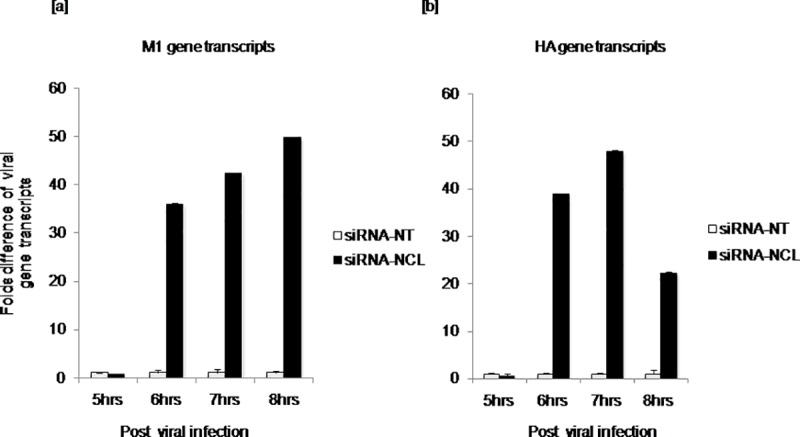
Influenza A viral late genes expression in cells transfected with siRNA-NCL or siRNA-NT. A549 cells were transfected with siRNA constructs followed by virus infections. At 5, 6, 7 and 8hrs post infection, total RNA was isolated and reverse transcribed. Complimentary DNA was subjected to quantitative real time PCR amplification using specific primers for viral genes encoding structural proteins; Matrix (M1) and Hemagglutinin (HA). Fold difference of gene expression between groups was done by calculating ‘n’ fold difference in mRNA abundance by 2^-ΔΔCT^ method and plotted as bar graph. The means of triplicate ΔC_T_ values for each of the viral gene expressed in cells with siRNA-NT or siRNA-NCL were compared by student’s t test. [a] M1 expression level at 6 to 7hrs (p ≤0.0001) and at 8hrs (p ≤0.002) [b] HA expression at 6 to 7hrs (p ≤0.0001) and at 8hrs (p ≤0.008) post infection.

Interaction of nucleolin and NP at 6hrs post infection is thought to be an early event in virus life cycle, and is expected to affect the subsequent steps of replication. Thus, to determine the impact of nucleolin-NP interaction on late phase of infection, expression of viral genes encoding structural proteins (late proteins); Matrix (M1) and Hemagglutinin (HA) was measured at transcript level by quantitative real time PCR. A549 cells transfected with siRNA-NCL or siRNA-NT were infected with virus following 24hrs transfection, viral RNA isolated at 5, 6, 7 and 8hrs post infection was reverse transcribed and subjected to real time PCR amplification using specific primers to M1 and HA genes. Fold difference of gene expression between groups at different time points was shown as bar graph. Interestingly, about 35 to 50fold increase of M1 gene expression at 6 to 7hrs (p≤0.0001) and at 8hrs (p≤0.002) was observed in cells with depleted endogenous nucleolin (Fig 5A). Similarly, significantly increased HA gene expression was noted at 6–7hrs (p≤0.0001), 8hrs (p≤0.008) in cells with siRNA-NCL when compared to cells with control construct siRNA-NT (Fig 5B). However, no obvious change was observed in M1 and HA gene transcripts level at 5hrs post infection. Significantly increased expression of late viral gene transcripts in cells with depleted endogenous nucleolin indicates the negative impact of this interaction on virus life cycle.

Subsequent experiments were carried out to measure M1, HA and NP gene transcripts at 24hrs post infection following transfection with siRNA constructs. Results obtained were comparable to the above experiment. M1 gene expression in cells with siRNA-NCL was significantly greater than in cells with siRNA-NT (p ≤0.0001) and HA gene expression was also observed to be significantly increased (p ≤0.0001). While no significant difference of NP (p = 0.27) gene expression was observed in siRNA-NCL *vs*. siRNA-NT at 24hrs post infection.

**Fig 6:**
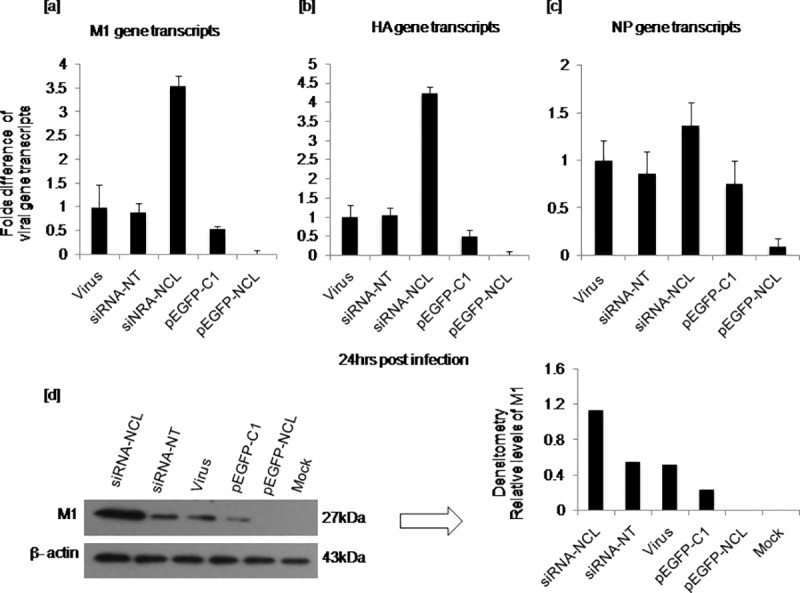
Comparison of viral late gene transcripts, M1, HA and early gene NP in cells transfected with siRNA-NCL and pEGFP-NCL. Transfections and infections were carried out as described in the previous experiment. In addition to transfections with siRNA constructs, cells were transfected with pEGFP-NCL or pEGFP-C1 vector followed by virus infection. At 24hrs post infection, total RNA was isolated, reverse transcribed and the complementary DNA was subjected to real time PCR amplification using specific primers. Fold difference among different groups was shown as bar graph. The means of triplicate ΔC_T_ values for each of the viral gene expressed in cells with different constructs were compared by student’s t test. M1 expression at protein level was measured in all the groups at 24hrs post infection following transfection with the desired constructs by western blotting using anti-viral M1 antibody. [a] M1 expression in cells with siRNA-NCL (p ≤0.0001), pEGFP-NCL (p ≤0.0001) [b] HA expression in cells with siRNA-NCL (p ≤0.0001) and pEGFP-NCL (p ≤0.0001) [c] NP expression in cells with siRNA-NCL (p = 0.27), pEGFP-NCL (p ≤0.0001) as compared to expression in cells with their corresponding controls; siRNA-NT and pEGFP-C1 [d] Viral M1 expression at protein level with the corresponding densitometry analysis.

To further investigate whether overexpression of nucleolin would circumvent the effects observed on late viral gene expression under depleted nucleolin conditions, cells were transiently transfected with pEGFP-NCL or pEGFP-C1(empty vector) followed by virus infections. Overexpression of nucleolin demonstrated significantly reduced late viral M1 (p ≤0.0001) (Fig 6A), HA (p ≤0.0001) (Fig 6B) and NP (p ≤0.0001) (Fig 6C) gene expression as compared to its control; cells with pEGFP-C1 vector. Similarly, expression of M1, HA and NP in cells with depleted nucleolin (siRNA-NCL) *vs*. over expressed nucleolin (pEGFP-NCL) was observed to be statistically significant (p ≤0.0001). However, no significant difference of expression of M1, HA and NP genes was noted in untransfected cells infected with virus *vs*. siRNA- NT or pEGFP-C1 cells. Corresponding polypeptide levels of M1 gene was measured by western blotting (Fig 6D) using anti-viral M1 antibody and the results correlated with that of transcript level.

Further, the significance of altered HA expression following altered endogenous nucleolin was determined by measuring the HA titer. HA is the late viral envelope glycoprotein and has the ability to agglutinate RBCs. To detect the presence of virus in cell lysates collected at 24hrs post infection following transfection with different constructs or in untransfected virus and mock infected cells, HA titer was measured. Two fold serially diluted viral lysates collected from each group was incubated with 50μl of 0.75% guinea pig RBCs for 1hr at RT. Viral lysate harvested at 72hrs post-infection from MDCK cells infected with Influenza A H1N1 pandemic virus was used as a positive control. Agglutinated RBCs were observed up to 1:4 diluted viral lysate recovered from cells with siRNA-NCL, while no agglutination was observed in lysate collected from cells with pEGFP-NCL (S4 Fig). This result supports the real time PCR data, and it is clear that overexpression of nucleolin significantly decreased the HA gene expression subsequently the formation of viral progeny.

### Effect of nucleolin and nucleoprotein interaction on the outcome of virus progeny particles

**Fig 7:**
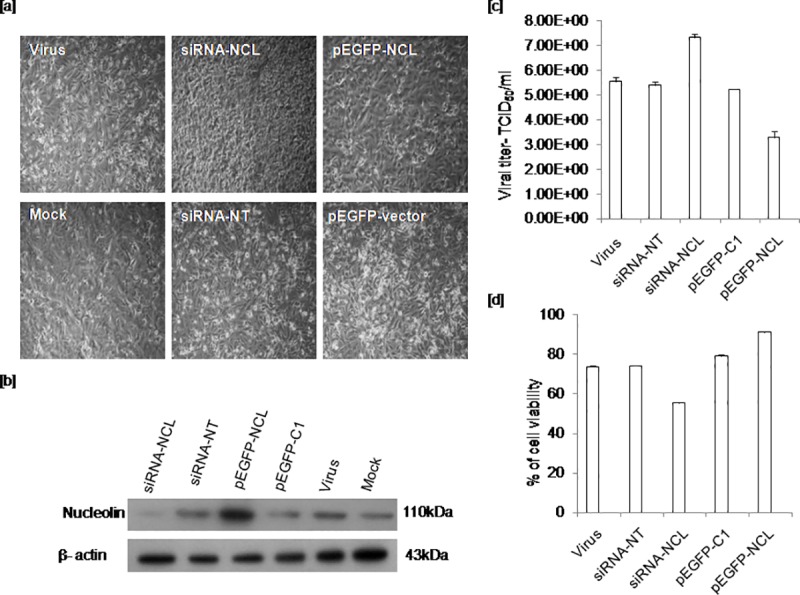
Virus induced morphological changes and titers in cells with depleted or over expressed nucleolin. A549 cells were transfected with siRNA-NCL or siRNA-NT or pEGFP-NCL or pEGFP-C1 constructs followed by infections. Untransfected but virus or mock infected cells were also included as controls. Infected cells were incubated at 37°C, 5% CO_2_ incubator and CPE was recorded at 48hrs post infection. Cells were harvested and the titer of the virus formed under depleted or overexpressed nucleolin condition was determined using TCID_50_ end point assay. A549 cells were further infected with serially diluted virus progeny collected from cells transfected with different constructs as mentioned above. At 72hrs post incubation, titer and the percentage of cell viability was measured. Means of triplicate values obtained for viral titers and the cell viability assay was compared by non parametric Mann-Whitney U test and student’s t test respectively. [a] Photomicrographs showing CPE in infected cells following transfection with different constructs [b] Nucleolin expression in cells transfected with each of the construct and in untransfected cells [c] Corresponding viral titers in cells with siRNA-NCL (p ≤0.0001), pEGFP- NCL (p ≤0.0001) as compared to siRNA-NT, pEGFP-C1 respectively [d] Percentage of cell viability in siRNA-NCL (p ≤0.0001), pEGFP-NCL (p ≤0.0001) as compared to controls. No significant difference of titer and cell viability were observed when comparison made among untransfected cells, siRNA-NT and pEGFP-C1 controls.

Altered expression of late viral genes following manipulation of nucleolin suggests that the interaction between nucleolin and viral NP early during infection may determine the outcome of viral progeny. To examine the effects of altered host cellular nucleolin expression on replication efficiency of virus, 50% endpoint virus titer was measured. A549 cells were transiently transfected with siRNA-NCL or siRNA-NT or pEGFP-NCL or pEGFP-C1 followed by virus infections. The morphological changes induced in cells transfected with siRNA-NCL or pEGFP-NCL were compared with their corresponding controls; siRNA-NT and pEGFP-C1 respectively. Strong CPE was observed in cells with siRNA-NCL while cells with siRNA-NT or pEGFP-C1 had shown moderate effect and the changes were comparable to untransfected but infected cells. However, the morphology of cells with pEGFP-NCL was reasonably similar to mock infected cells (Fig 7A) with no visible CPE. Nucleolin expression in A549 cells with different constructs was measured by western blotting (Fig 7B). Cells were harvested at 48hrs infection following 24hrs transfection and infectious titer of virus progeny formed in cells with reduced (siRNA-NCL) or overexpressed (pEGFP-NCL) or standard (siRNA-NT/pEGFP-C1/untransfected) nucleolin level was determined by TCID_50_. A549 cells were further infected with serial dilutions (log dilutions) of virus lysates collected from cells transfected with above constructs independent of each other. Infected cells were incubated and monitored for CPE. At 72hrs post infection, CPE was recorded and infectious titer was measured. Viral titer was measured in three independent experiments. Means of triplicate TCID_50_ values were compared by non parametric, Mann-Whiteny U test. In accordance with late viral gene expression, significantly increased viral titer was recorded in cells with siRNA-NCL (p ≤0.0001) as compared to siRNA-NT cells. Wherein, cells with pEGFP-NCL (p ≤0.0001) had demonstrated significantly reduced viral titer as compared to its control cells with pEGFP-C1 vector. Statistically significant difference in viral titer was observed in cells with siRNA-NCL and pEGFP-NCL (p ≤0.0001) (Fig 7C). No significant difference in titer was noted among infected cells with control plasmids; siRNA-NT, pEGFP-C1 and untransfected cells. Similar experiment was carried out one more time and the titer of the released infectious viral progeny was measured in the medium collected from each group of cells. Resulting titers were comparable with the titers of cell associated and cell free virus assayed together (S5 Fig).

Percentage of cell viability in the above set of cells as determined by MTT assay had demonstrated identical results with increased cell death under nucleolin depleted conditions (siRNA-NCL) (p ≤0.0001). High percentage of cells remained viable in cells with overexpressed nucleolin (pEGFP-NCL) (p ≤0.0001) when compared to its control (Fig 7D). Our experimental evidences suggest that downregulation of host nucleolin facilitates the virus to undergo multiple rounds of replication resulting in increased viral progeny.

### Nucleolin interaction with the viral nucleoprotein is common among seasonal strains of influenza A virus

**Fig 8:**
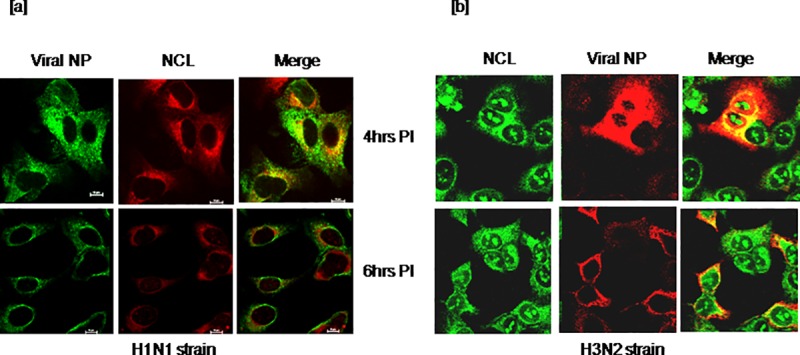
Validation of host nucleolin and viral nucleoprotein interaction in cells infected with influenza A viral seasonal H1N1 and H3N2 strains. A549 cells grown on cover slips were infected with seasonal strains as described earlier. At every 2hrs intervals up to 10hrs cells were fixed and subjected to double IFA staining and analyzed by confocal microscopy. Co-localization of viral NP and host nucleolin at 4–6hrs in cells infected with [a] H1N1 [b] H3N2 strain (Contrary to H1N1 staining, secondary antibody with TRITC was used to stain viral NP and FITC to stain viral NP in cells infected with H3N2).

All the above experiments were carried out using influenza A virus of 2009 pandemic H1N1 strain. To investigate whether nucleolin interaction with viral NP is common among seasonal strains or specific to pandemic strain used in the current study, A549 cells were infected with influenza A virus seasonal strains, H3N2 and H1N1 which are known to circulate among humans. At different time points, infected cells were analyzed by double IFA staining. Co-localization of host nucleolin with viral NP in the cytoplasm of infected cells at 4 to 6hrs post infection (Fig 8A and 8B) substantiates that the nucleolin is a common host interacting partner to NP of pandemic and seasonal strains.

## Discussion

Host and viral protein interactions are crucial for virus to replicate and establish infection in a susceptible host. In the current study, we identified host nucleolin as a novel interacting partner to recombinant influenza A viral NP by co-immunoprecipitation assay followed by MALDI-TOF/MS analysis. Interaction of two proteins was validated in A549 cells infected with the corresponding 2009 pandemic H1N1 strain. Host nucleolin and viral NP interaction was observed early during infection; about 6hrs following viral adsorption. The association was noted prominently in the cell cytoplasm. Disruption of nucleolin-NP interaction following depletion of endogenous nucleolin with siRNA-NCL construct confirmed the above finding. Having confirmed the interaction of two proteins, its impact on subsequent steps of viral replication was investigated. As the association of two proteins was observed early during infection, late viral gene expression and viral titers were measured in infected cells under nucleolin depleted or overexpressed conditions. Interestingly, depletion of endogenous nucleolin significantly increased the late viral gene expression and titer while overexpression of nucleolin significantly reduced the gene expression resulting in reduction of infectious viral progeny. These two experiments together suggest that the interaction of host nucleolin with viral NP during early phase of infection is an unfavorable event and interrupts the virus life cycle. Nucleolin is reported to be involved in the infection process of RNA and DNA viruses and it plays a significant role in various stages of replication and pathogenesis [31, 32].

Nucleolin, a multifunctional eukaryotic protein is ubiquitously distributed in a cell. It plays an important role in pre-rRNA transcription, transcriptional elongation and ribosome assembly [33]. It is one of the most abundant proteins of nucleolus and has 710 amino acids with an approximate molecular weight of 76kDa. Due to unique phosphorylation sites in its N-terminus domain, it is usually detected in cells in phosphorylated form with an apparent molecular weight of 110kDa [34, 35]. Interaction of nucleolin with other cellular proteins is mediated through its N- terminal domain [36]. It shuttles between nucleolus and cytoplasm [37], plasma membrane [38] and it has been implicated in many cellular functions such as gene silencing, cell cycle regulation etc., [39, 40]. It largely locates to the cell nucleolus and certain viruses known to hijack the dynamic nucleolus to establish the infection in the host cell [31]. In general RNA viruses replicate inside the cytoplasm, but several studies demonstrate interaction of RNA viruses with nucleolin within the nucleolus. Following initiation of viral replication, host nuclear components including nucleolus are morphologically changed. Nucleolar change in response to influenza virus interaction with nucleolin was first time demonstrated in chick embryo fibroblasts [41]. It was also reported to interact with viruses when it relocates to the cytoplasm or to the plasma membrane. Poliovirus is one among them, which replicates inside the cytoplasm of a susceptible cell. During poliovirus infection, nucleolin relocates to cytoplasm and interacts with the 5’-non coding region of viral genome; internal ribosomal entry site to initiate the translation of non-capped viral mRNA [42]. It is known to interact with NS5B of HCV, another RNA virus through two independent regions of NS5B protein [43]. This observation was supported by another study where truncated nucleolin with retained HCV NS5B binding sites has shown to inhibit the RNA-dependent RNA polymerase activity of NS5B [44]. Similarly, a significant role of nucleolin was demonstrated in the life cycle of Hepatitis D virus. Through its interaction with two isoforms of Hepatitis D antigens of different sizes regulate the replication and assembly respectively [45]. Nucleolin interacts with other RNA viruses such as parainfluenza type 3 virus during viral entry [46], rabies virus phosphoprotein during its replication [47], and with dengue virus capsid protein it facilitates the formation of infectious virus particles [48]. It is evident from the above studies that nucleolin regulates multiple functions during infection process of a large number of viruses through its interaction with non-structural and structural proteins.

Unlike other RNA viruses, influenza virus replicates inside the nucleus following the import of RNP complexes to the nucleus. It hijacks the host nuclear machinery and induces strong remodeling of the host nucleus ultrastructure, nucleolus being an early target [31]. Association of nuclear protein such as importin β3 and PARP1 with the polymerase complex and association of DNA damage binding protein and nucleophosmin (NPM) with vRNA were demonstrated [14]. Interaction of nucleolin with influenza A viral non structural protein (NS1) and envelope glycoprotein hemagglutinin (HA) was also reported. NS1 protein of influenza A viral H3N2 strain does interact with the nucleolin within the nucleolus during early stage of infection [49]. Later it was demonstrated that H3N2 strain interacts primarily via the c-terminal NLS2/NoLS and to a minor extent via the N-terminal NLS1 with the main nucleolar proteins; nucleolin, B23 and fibrillarin [50]. However, the significance of this interaction is not known. A recent study demonstrated the specific binding of cell surface nucleolin with viral envelope glycoprotein HA and its significant role in virus internalization using H1N1 strain. The mechanism was shown relatively conserved among other influenza virus strains, H5N1 and H7N9 [51]. Interaction of host nucleolin with NS1 [49, 50], HA protein [51] irrespective of the strain and site of interaction has demonstrated to facilitate the viral replication. On contrary to their findings, here, the identified novel interaction of NP of 2009 pandemic H1N1 strain with host nucleolin in the cytoplasm does not appear to be favorable to the virus. Manipulation of cellular nucleolin level resulted in an altered expression of late viral genes and consequently infectious viral progeny production. Unfavorable interaction between the nucleolin and various viral proteins is not uncommon. Direct interaction of retroviral *gag* protein with nucleolin inhibits the viral assembly [52]. Interaction of human replication protein A (hRPA) with SV-40 initiation complex is essential for productive infection, but nucleolin interaction with hRPA inhibits the unwinding of the origin of SV-40 genome and its replication in the host cell [53].

Convergent results from independent studies including our study demonstrated diverse functions following interaction of nucleolin with influenza A virus proteins during replication. The diverse functions are found to be strain specific. Influenza A virus seasonal strains have been used to show the effects of host nucleolin and virus-protein interaction in reported studies. However, 2009 pandemic H1N1 strain of swine origin was used in the current study. Melen et al had demonstrated that, influenza A viral NS1 protein interaction with nucleolin is strain specific [50]. Likewise, inhibition of nucleolin had been reported to be common among different influenza A viral subtypes but the efficiency of its inhibition on virus internalization was observed to be different among subtypes [51]. Moreover, the localization of nucleolin also appears to be important for its association with the viral protein. Multiple functions of host nucleolin are achieved through complex formation with other proteins including viral proteins in infected cells. Complex formation may take place in different cellular compartments, as nucleolin is found in the cytoplasm and cell surface independent of nucleolar localization. Further, post translational modifications such as phosphorylation, acetylation, methylation and glycosylation relates to its localization as the membrane bound nucleolin is glycosylated [54] but not the nucleolar nucleolin. So, the viral strain infecting the cell and the localization of nucleolin following its post translational modifications together may play a significant role in determining the destination of nucleolin-viral protein interaction and the degree of pathogenesis. Influenza virus NP share certain similarities with host nucleolin such as the ability to shuttle between nucleus and other cellular compartments and both the proteins are known to have RNA binding domains. Hence, it is essential to comprehend the contribution of these characteristics for interaction with each other in the cytoplasm during early phase of infection. Considering the importance of nucleolin interaction with different influenza A virus proteins of different strain origins, the host and viral factors that determine the localization of viral protein interaction with host nucleolin is worth exploring.

To conclude, host nucleolin is a novel partner to influenza A virus NP of 2009 pandemic H1N1 strain. Based on our preliminary data, it is enticed to speculate that the host nucleolin through its interaction with viral NP in the cell cytoplasm possibly restricts the nuclear import of NP to initiate the replication. As expected, the level of nucleolin was observed to be high in cytosolic fraction of infected cells as compared to mock infected cells (S2 Fig). In addition, accumulation of viral NP in cytosolic fraction as compared to nuclear fraction supports the above speculation. It is evident from our study that nucleolin interaction with viral NP is possibly a host defense mechanism as the altered nucleolin levels altered the late viral gene expression and titers. But the recorded viral titer in control cells suggests that the virus is able to overcome the initial effect imposed by the host through nucleolin-NP interaction and thus efficiently produce the infectious viral progeny. Nonetheless, the accumulation of nucleolin in the cytoplasm during early phase of infection and the molecular mechanism underlying this interaction need to be investigated in greater detail to aim for anti-viral therapeutics to target this interaction.

While this paper was under revision, a complementary paper describing the host nucleolin interaction with influenza A viral nucleoprotein was published (Terrier O, Carron C, De Chassey et al,. Nucleolin interacts with influenza A nucleoprotein and contributes to viral ribonucleoprotein complexes nuclear trafficking and efficient influenza viral replication. Scientific Reports 6.29006, DOI: 10.1038/srep29006). However, using H3N2 seasonal strain they demonstrated that nucleolin-NP interaction contributes to viral replication, which is in contrast to our finding that shows the restriction of late viral gene expression following nucleolin interaction with NP of 2009 pandemic H1N1 strain. This could be due to nucleoprotein of different strain origins used. Various influenza A strains may have common interacting host partners, but the effects exerted are possibly being strain specific.

## Supporting Information

S1 Fig Co-localization of host nucleolin and recombinant viral NP. A549 cells grown on coverslips were transiently transfected with the recombinant viral NP or the empty vector. Cells fixed 24hrs post transfection were subjected to double IFA staining using anti-nucleolin, anti-Myc antibodies and secondary antibodies with two fluorophores. Images show co-localization of recombinant viral NP and host nucleolin in two individual cells from a single field. Co-localization coefficients of two cells as determined by PCC and MOC are 0.68, 0.83 and 0.83, 0.89 respectively.
(TIF)

S2 Fig Endogenous nucleolin expression in subcellular fractions of infected cells. A549 cells were infected with influenza A virus and mock infected with serum free medium. At 4 to 8hrs post infection, at every 1hr interval, cells were harvested and subcellular fractionation was done. Endogenous nucleolin expression in cytosolic and nuclear fractions was measured by western blotting using anti-nucleolin antibody and the viral NP expression using anti-viral NP antiserum. [a] Nucleolin and NP expression in two fractions of virus infected cells [b] Nucleolin expression in two fractions of mock infected cells. Marker proteins; β-actin and c-Jun expression confirmed the purity of cytoplamic and nuclear fractions prepared from virus and mock infected cells.
(TIF)

S3 Fig Optimization of *in vitro* binding of recombinant viral NP and host nucleolin. BL-21 cells were transformed with the recombinant viral NP (pET29a+NP) or unrelated control protein and cell lysates were prepared. Either 100μg of bacterial lysate incubated with different concentrations of Ni-NTA beads ranging from 12.5 to 100μl or 100μl of beads incubated with different concentrations of bacterial lysate ranging from 50 to 250μg for 6hrs to immobilize the recombinant protein on Ni-NTA beads. Further, beads were washed and incubated with 1mg of A549 cell lysate. Next day, after washing the beads, bound protein complexes were eluted and subjected to SDS PAGE followed by immunoblotting with anti-nucleolin and anti-His antibodies. Cell lysates recovered after centrifugation following incubation with recombinant viral NP and control protein bound Ni-NTA beads were analyzed for endogenous nucleolin expression. [a] Binding of 110kDa nucleolin protein and the recombinant viral NP with the use of 100μl beads [b] Dose dependent binding of nucleolin with viral NP [c] and [d] No visible binding of nucleolin with the control protein. Expression of recombinant viral NP, control protein and nucleolin was shown in the corresponding bacterial and A549 cell lysates.
(TIF)

S4 Fig Influenza A viral hemagglutination assay (HA assay). HA titer was measured in virus lysates harvested at 24hrs post infection from A549 cells transfected with siRNA-NCL or siRNA-NT or pEGFP-NCL or pEGFP-C1. Viral lysates recovered from untransfected but virus infected and mock-infected cells at 24hrs post infection were used as controls. Twofold serial dilutions of each sample was made in 1× PBS and incubated with guinea pig RBCs. Agglutination of RBCs was recorded for each sample. HA assay showing agglutination by virus lysate collected from siRNA-NCL cells up to 1:4 dilutions. No visible agglutination was observed by pEGFP-NCL cell lysate.
(TIF)

S5 Fig Titer of infectious viral progeny released from cells with depleted and overexpressed nucleolin. A549 cells were transfected with siRNA-NCL or siRNA-NT or pEGFP-NCL or pEGFP-C1 constructs followed by infections. Untransfected but virus or mock infected cells were included as controls. At 48hrs post infection following 24hrs transfection, medium from infected cells was collected and the titer of the released infectious viral progeny in each sample was determined by TCID_50_ assay as described in Fig 7.
(TIF)
