# Supplementary figures and images for: Interaction of Host Nucleolin with Influenza A Virus Nucleoprotein in the Early Phase of Infection Limits the Late Viral Gene Expression
Source: PLoS One. 2016 Oct 6;11(10):e0164146. doi: 10.1371/journal.pone.0164146 (PMC5053498; doi:10.1371/journal.pone.0164146)

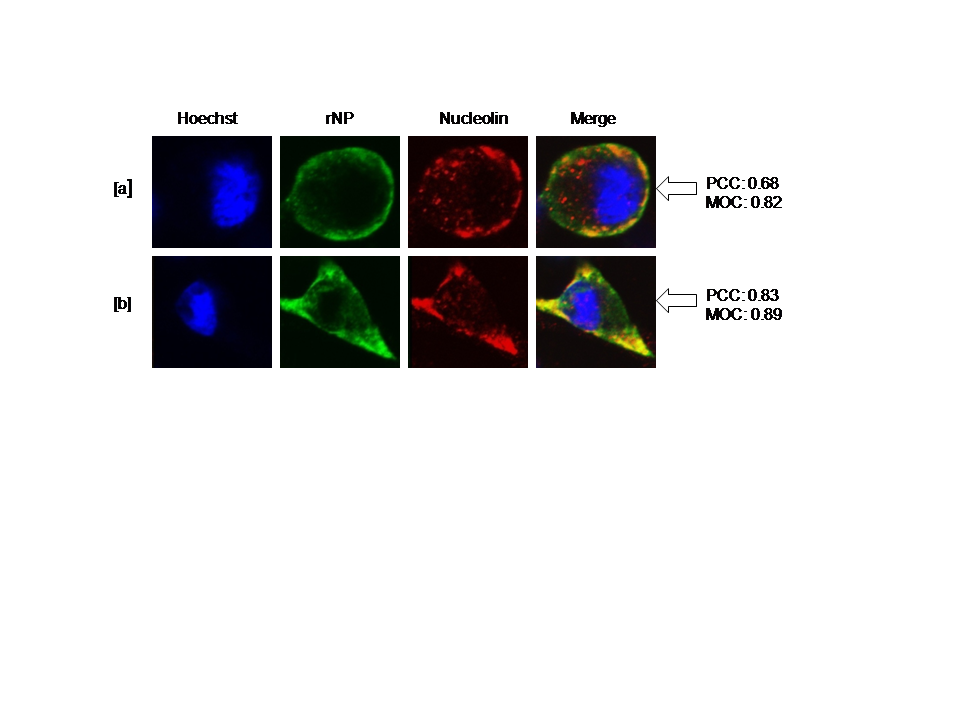

Supplement: S1 Fig — A549 cells grown on coverslips were transiently transfected with the recombinant viral NP or the empty vector. Cells fixed 24hrs post transfection were subjected to double IFA staining using anti-nucleolin, anti-Myc antibodies and secondary antibodies with two fluorophores. Images show co-localization of recombinant viral NP and host nucleolin in two individual cells from a single field. Co-localization coefficients of two cells as determined by PCC and MOC are 0.68, 0.83 and 0.83, 0.89 respectively. (TIF) [file pone.0164146.s001.tif]

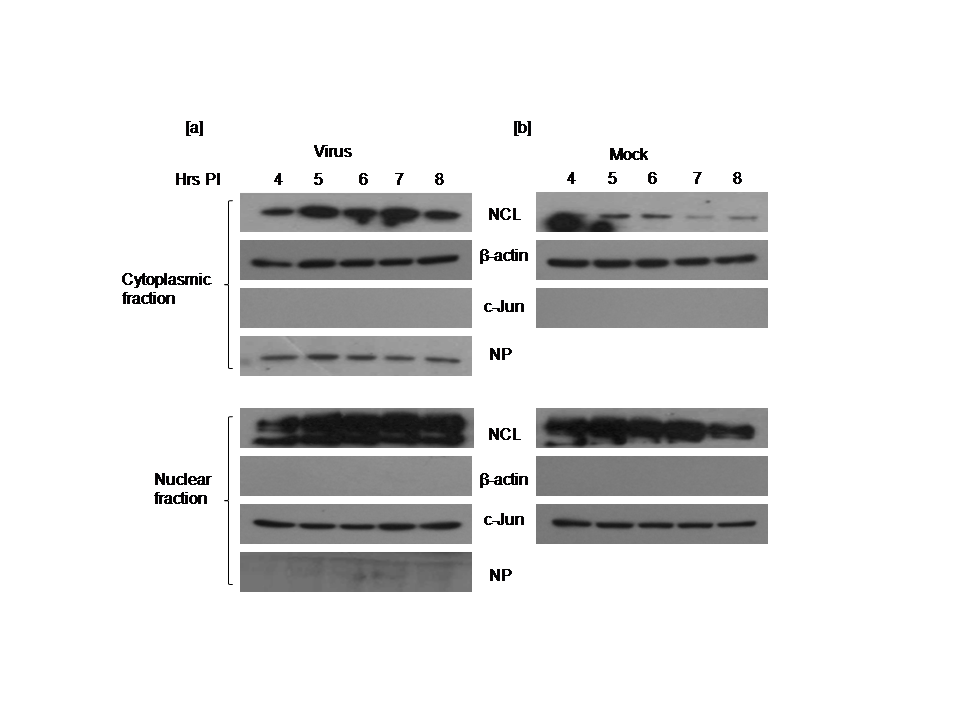

Supplement: S2 Fig — A549 cells were infected with influenza A virus and mock infected with serum free medium. At 4 to 8hrs post infection, at every 1hr interval, cells were harvested and subcellular fractionation was done. Endogenous nucleolin expression in cytosolic and nuclear fractions was measured by western blotting using anti-nucleolin antibody and the viral NP expression using anti-viral NP antiserum. [a] Nucleolin and NP expression in two fractions of virus infected cells [b] Nucleolin expression in two fractions of mock infected cells. Marker proteins; β-actin and c-Jun expression confirmed the purity of cytoplamic and nuclear fractions prepared from virus and mock infected cells. (TIF) [file pone.0164146.s002.tif]

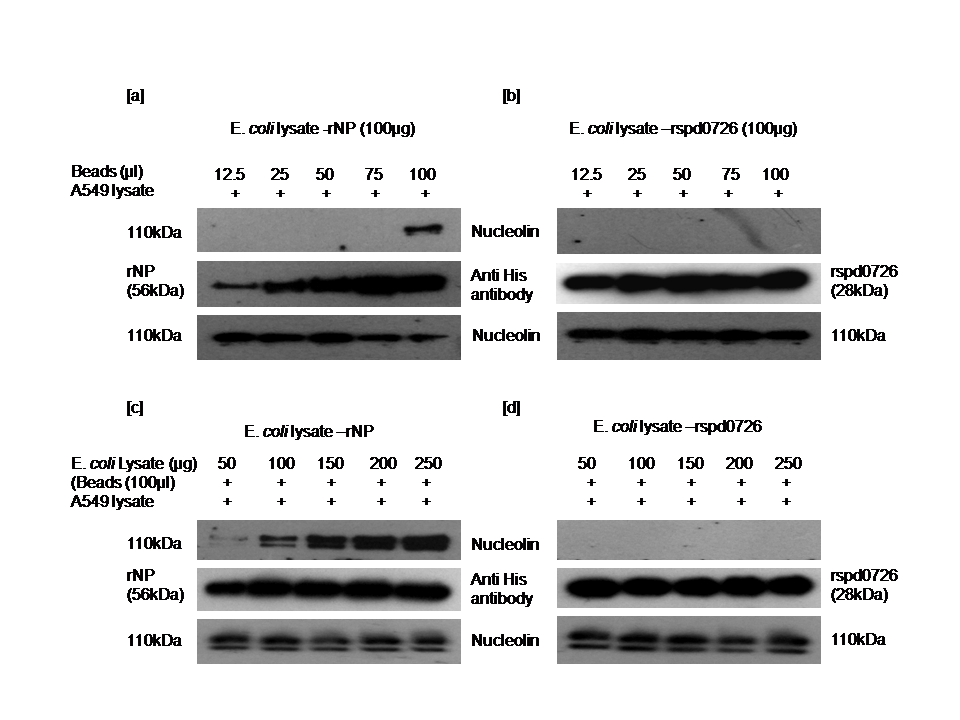

Supplement: S3 Fig — BL-21 cells were transformed with the recombinant viral NP (pET29a+NP) or unrelated control protein and cell lysates were prepared. Either 100μg of bacterial lysate incubated with different concentrations of Ni-NTA beads ranging from 12.5 to 100μl or 100μl of beads incubated with different concentrations of bacterial lysate ranging from 50 to 250μg for 6hrs to immobilize the recombinant protein on Ni-NTA beads. Further, beads were washed and incubated with 1mg of A549 cell lysate. Next day, after washing the beads, bound protein complexes were eluted and subjected to SDS PAGE followed by immunoblotting with anti-nucleolin and anti-His antibodies. Cell lysates recovered after centrifugation following incubation with recombinant viral NP and control protein bound Ni-NTA beads were analyzed for endogenous nucleolin expression. [a] Binding of 110kDa nucleolin protein and the recombinant viral NP with the use of 100μl beads [b] Dose dependent binding of nucleolin with viral NP [c] and [d] No visible binding of nucleolin with the control protein. Expression of recombinant viral NP, control protein and nucleolin was shown in the corresponding bacterial and A549 cell lysates. (TIF) [file pone.0164146.s003.tif]

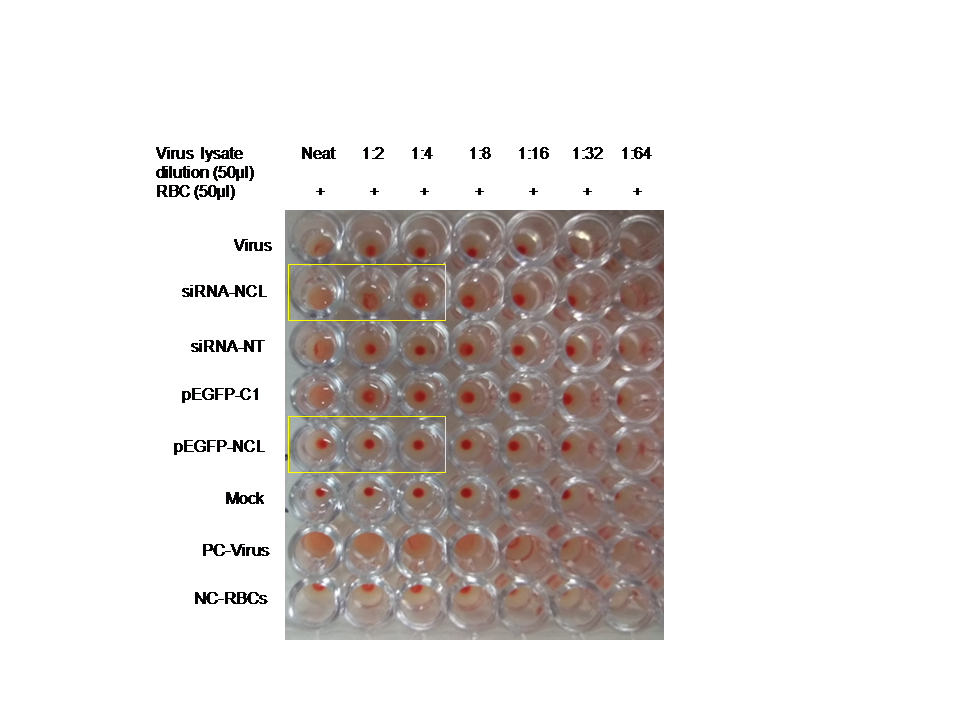

Supplement: S4 Fig — HA titer was measured in virus lysates harvested at 24hrs post infection from A549 cells transfected with siRNA-NCL or siRNA-NT or pEGFP-NCL or pEGFP-C1. Viral lysates recovered from untransfected but virus infected and mock-infected cells at 24hrs post infection were used as controls. Twofold serial dilutions of each sample was made in 1× PBS and incubated with guinea pig RBCs. Agglutination of RBCs was recorded for each sample. HA assay showing agglutination by virus lysate collected from siRNA-NCL cells up to 1:4 dilutions. No visible agglutination was observed by pEGFP-NCL cell lysate. (TIF) [file pone.0164146.s004.tif]

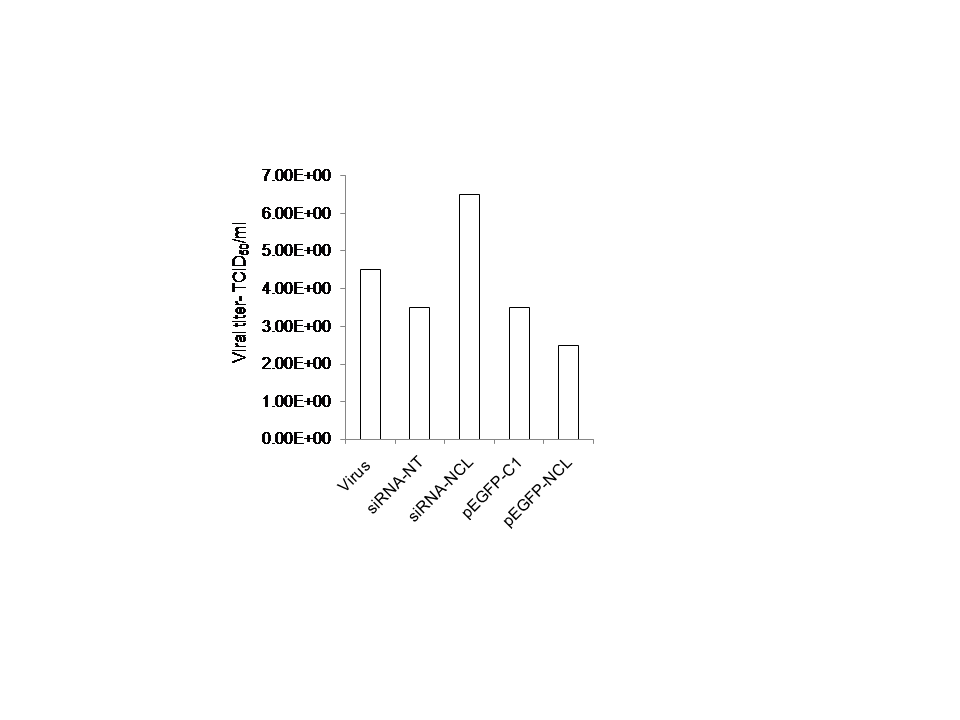

Supplement: S5 Fig — A549 cells were transfected with siRNA-NCL or siRNA-NT or pEGFP-NCL or pEGFP-C1 constructs followed by infections. Untransfected but virus or mock infected cells were included as controls. At 48hrs post infection following 24hrs transfection, medium from infected cells was collected and the titer of the released infectious viral progeny in each sample was determined by TCID50 assay as described in Fig 7. (TIF) [file pone.0164146.s005.tif]
